# Evidence-based treatment for adult women with child abuse-related Complex PTSD: a quantitative review

**DOI:** 10.3402/ejpt.v5.23613

**Published:** 2014-10-14

**Authors:** Ethy Dorrepaal, Kathleen Thomaes, Adriaan W. Hoogendoorn, Dick J. Veltman, Nel Draijer, Anton J. L. M. van Balkom

**Affiliations:** 1 GGZ inGeest, Amsterdam, The Netherlands; 2 Department of Psychiatry, VU University Medical Center, Amsterdam, The Netherlands; 3 EMGO Institute, VU University Medical Center, Amsterdam, The Netherlands; 4 PsyQ, Parnassia Groep, The Hague, The Netherlands

**Keywords:** Review, meta-analysis, PTSD, psychotherapy, cognitive behavioral therapy, cognitive behavioral treatment, child abuse, childhood abuse, adult survivors of child abuse

## Abstract

**Introduction:**

Effective first-line treatments for posttraumatic stress disorder (PTSD) are well established, but their generalizability to child abuse (CA)-related Complex PTSD is largely unknown.

**Method:**

A quantitative review of the literature was performed, identifying seven studies, with treatments specifically targeting CA-related PTSD or Complex PTSD, which were meta-analyzed, including variables such as effect size, drop-out, recovery, and improvement rates.

**Results:**

Only six studies with one or more cognitive behavior therapy (CBT) treatment conditions and one with a present centered therapy condition could be meta-analyzed. Results indicate that CA-related PTSD patients profit with large effect sizes and modest recovery and improvement rates. Treatments which include exposure showed greater effect sizes especially in completers’ analyses, although no differential results were found in recovery and improvement rates. However, results in the subgroup of CA-related *Complex* PTSD studies were least favorable. Within the Complex PTSD subgroup, no superior effect size was found for exposure, and affect management resulted in more favorable recovery and improvement rates and less drop-out, as compared to exposure, especially in intention-to-treat analyses.

**Conclusion:**

Limited evidence suggests that predominantly CBT treatments are effective, but do not suffice to achieve satisfactory end states, especially in Complex PTSD populations. Moreover, we propose that future research should focus on direct comparisons between types of treatment for Complex PTSD patients, thereby increasing generalizability of results.

Effective treatments for posttraumatic stress disorder (PTSD) are well established: first-line treatments include several types of cognitive behavior therapy (CBT), such as prolonged exposure (PE), cognitive (processing) therapy (C(P)T) with and without exposure, and Eye Movement Desensitization and Reprocessing (EMDR) (Bradley, Greene, Russ, Dutra, & Westen, 2005; Cloitre, 2009). However, to date there is only sparse evidence for effective treatments in child abuse (CA)-related Complex PTSD. By interfering with normal development, CA may result in PTSD complicated by problems in affect regulation, memory and attention, self-perception, interpersonal relations, somatization, and systems of meaning (Herman, 1992). This syndrome is referred to as “PTSD with associated features” in DSM-IV-TR (APA, 2000) or “Complex PTSD,” and is characterized by high comorbidity on both DSM-IV Axis I and II. Empirical studies as well as neurobiological findings support the distinction between Complex PTSD and DSM-defined PTSD (Ford, 1999; Lanius et al., 2010; Thomaes et al., 2010, 2013; Van Der Kolk, Roth, Pelcovitz, Sunday, & Spinazzola, 2005; Zlotnick et al., 1996). A prevalence of 1% of Complex PTSD has been observed in a student population (Ford, Stockton, Kaltman, & Green, 2006).

Reviews on CA (with a diversity of symptoms, not specifically PTSD) (Callahan, Price, & Hilsenroth, 2004; Kessler, White, & Nelson, 2003; Martsolf & Draucker, 2005; Peleikis, Mykletun, & Dahl, 2005; Taylor & Harvey, 2010) showed that a variety of treatments can be beneficial. Earlier reviews mainly included group treatments, while more recently individual treatments showed favorable effect sizes. Structured treatment characteristics such as availability of a manual, an instructional format and providing homework increased treatment effect in terms of PTSD symptoms, while externalizing problems were unaffected (Taylor & Harvey, 2010). However, these reviews included a limited number of randomized controlled trials (RCTs) with adequately diagnosed PTSD, and these PTSD studies were not analyzed separately to investigate differential treatment effects. Therefore, generalizing these results to the CA-related Complex PTSD population is problematic.

Reviews on PTSD (resulting from various trauma types, not specifically CA) concluded that active treatments for PTSD are highly effective and superior to waiting list (WL) controls (Benish, Imel, & Wampold, 2008; Bisson et al., 2007; Bradley et al., 2005; Cloitre, 2009; Powers, Halpern, Ferenschak, Gillihan, & Foa, 2010; Schottenbauer, Glass, Arnkoff, Tendick, & Gray, 2008; Seidler & Wagner, 2006). The largest body of evidence has been accumulated for CBT, either exposure, cognitive therapy, or both, and EMDR. These reviews included only a small number of primary RCTs concerning CA populations, again limiting generalizability to the CA-related Complex PTSD population. Powers et al. (2010) found no significant difference in effect sizes between studies with and without a child sexual abuse population, based on two (partly) CA studies. However, this non-finding could be explained by the fact that they reported neither on exposure to other complex trauma, nor on the presence or absence of Complex PTSD. In women with a history of CA and chronic interpersonal violence, Cloitre (2009) found a range of CBT treatments effective to achieve PTSD symptom reduction. In addition, a few treatments were reported that focused on other symptom domains, such as affect management (AM) and interpersonal skills training, with beneficial results in these domains as well.

To our knowledge, reviews which focus exclusively on the efficacy of treatments of CA-related Complex PTSD or CA-related PTSD are sparse. Furthermore, these reviews show considerable differences in study selection (target population, study design), outcome measures and data analysis methods (effect size, recovery and/or improvement rates, intention-to-treat analysis or completers’ analysis) (Peleikis & Dahl, 2005; Price, Hilsenroth, Petretic-Jackson, & Bonge, 2001). Moreover, it is doubtful whether the results of these reviews can be generalized to the CA-related Complex PTSD population, since only a few studies on both CA and PTSD were included, and little attention was paid to indicators of complexity such as Axis II comorbidity. Also, exclusion criteria such as suicidality and self-injurious behavior may have resulted in the exclusion of Complex PTSD patients. Thus, drawing conclusions based on the currently available empirical evidence for effective treatments in CA-related Complex PTSD is problematic. Consequently, it is still unclear for clinicians whether Complex PTSD patients are generally able to tolerate, and benefit from, commonly available first-line treatments equally well as DSM-defined PTSD patients, and opinions are divided on this issue. Complex PTSD as well as PTSD with Borderline Personality Disorder (BPD) has been associated with poor treatment outcome (Cloitre & Koenen, 2001; Ford & Kidd, 1998) and a higher drop-out rate following exposure (Cloitre et al., 2010; McDonagh et al., 2005). Moreover, first-line PTSD treatments may not target all relevant pathology in the CA population, such as poor affect regulation and interpersonal problems.

Summarizing, after early severe CA, DSM-defined PTSD may be complicated by additional features referred to as Complex PTSD. Reviews on CA as well as reviews on DSM-defined PTSD conclude that effective treatments are available for CA or PTSD, but research on the overlap between these populations is scarce, and generalizability of results to the CA-related *Complex* PTSD population is questionable. Moreover, treatment effects and compliance of different types of treatments with varying duration, structure and content in CA-related *Complex* PTSD are insufficiently known. Therefore, we aimed to investigate which evidence is available to effectively treat the subgroup of CA-related Complex PTSD. We define Complex PTSD as PTSD according to DSM-IV criteria plus Disorder of Extreme Stress (as measured by the SIDES; Van der Kolk et al., 2005), which is based on the WHO field trials on Complex PTSD in which it has been shown that 94–96% of Complex PTSD patients fulfill criteria of DSM-IV defined PTSD (Van der Kolk et al., 2005). This has led to the current proposal to categorize PTSD and Complex PTSD as sibling disorders in ICD-11, sharing the DSM-defined PTSD symptoms with the added symptom domains of (1) affect dysregulation, (2) negative self-concept, and (3) interpersonal disturbances in Complex PTSD (Cloitre, Garvert, Brewin, Bryant, & Maercker, 2013).

## Method

### Literature search

Our literature search covered the period January 1965 to May 2012. We searched MEDLINE using the following terms: CA OR childhood abuse OR child sexual abuse OR childhood physical abuse OR maltreatment OR PTSD OR posttraumatic OR DESNOS AND treatment OR therapy AND controlled trial OR clinical trial OR randomized OR review OR meta-analysis. These terms were searched as key words, title, abstract and Mesh terms. Findings were cross-referenced with references from reviews. We included published randomized original studies comparing interventions with other interventions or control conditions in study populations combining CA and PTSD (not only Complex PTSD) for comparison with other reviews on this issue. Inclusion criteria were (1) >50% of participants who met DSM-III-R, DSM-IV, or DSM-IV-TR criteria for posttraumatic stress disorder or PTSD as a main treatment target; (2) >50% of participants with CA or CA analyzed separately; (3) random assignment; (4) study participants at least 18 years of age; (5) the study had to test a specific psychotherapeutic treatment against a control condition and/or an alternative treatment; (6) the study had to be reported in English.

### Study selection

A total of 24 RCTs were identified that satisfied inclusion criteria, including both >50% patients with PTSD symptoms, as well as >50% patients with a history of CA.

These 24 studies were heterogeneous with regard to the number of patients meeting criteria for PTSD diagnosis or other PTSD indicators (e.g., level of PTSD symptoms), and the number of patients meeting criteria for Complex PTSD (as measured by the SIDES) or other Complex PTSD indicators (e.g., percentage comorbid personality disorders), and the number of patients with a CA history. Moreover, they differed in index trauma (CA or other trauma) and outcome measures. On the basis on these factors we established five categories:

- CA-related Complex PTSD (4 RCTs),
- CA-related PTSD (3 RCTs),
- All PTSD+mainly CA (9 RCTs),
- All CA+mainly with PTSD (5 RCTs),
- Mainly PTSD+mainly CA (3 RCTs).

Category A consists of studies on Complex PTSD as measured by the SIDES,^1,7^ and when SIDES ratings were not available we used studies in which patients met criteria for PTSD plus comorbid personality disorders, as a proxy for Complex PTSD.^5,6^ In this category, all patients were diagnosed with PTSD, all suffered from CA as the index trauma and the treatment target was Complex PTSD. In category B, all study participants were diagnosed with CA-related PTSD with some indicators covering Complex PTSD symptoms (e.g., dissociation, interpersonal problems, affect modulation, and anger), without classifying this as “Complex.” Studies assigned to category C focused on PTSD patients, of which the majority suffered from a CA history, but CA was not the index trauma in the majority of the patients. In category D, the patients had a CA history and CA was the index trauma; however, only parts of these patients were diagnosed with PTSD and this subgroup was not analyzed separately. Category E pertained to studies with not all but mainly patients suffering from CA as well as a mainly suffering from PTSD.

**Fig. 1:**
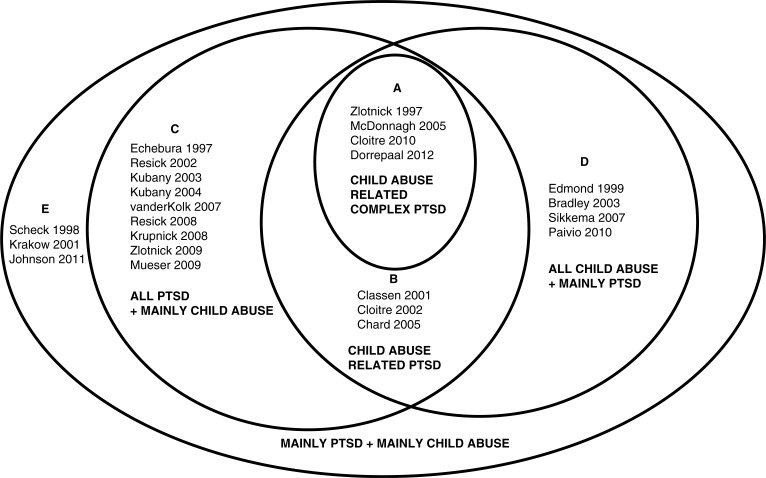
RCTs on child abuse (CA) and or PTSD. **Category A: CA-related Complex PTSD (4 RCTs):** All study participants diagnosed with Complex PTSD or >50% with personality disorder; PTSD as target symptoms. All CA. All CA as index trauma. **Category B: CA-related PTSD (3 RCTs):** All study participants diagnosed with PTSD as target symptoms. Outcome measures also covering some Complex PTSD symptoms All CA. All CA as index trauma. **Category C: All PTSD + mainly CA (9 RCTs):** All study participants diagnosed with PTSD as target symptoms. >50% CA or <50% but CA population analyzed separately. Index trauma sometimes CA, mainly rape or domestic violence. **Category D: All CA + mainly PTSD (5 RCTs):** Study participants >50% PTSD or substantial PTSD symptomatology (PTSD patients not analyzed separately); PTSD as target symptoms. All CA. All CA as index trauma. **Category E: Mainly PTSD + mainly CA (3 RCTs):** Study participants >50% PTSD (as target symptoms) and >50% CA.

Figure 1 is a diagram showing all RCTs grouped according to these criteria, in order to visualize their relevance in terms of our research question on evidence-based treatments for CA-related PTSD or Complex PTSD and in terms of index trauma (CA or other trauma) and outcome measures. Since we were specifically interested in treatments focusing on CA-related Complex PTSD, we selected the studies of category A and B, because these studies all included outcome measures covering Complex PTSD symptoms. We performed a separate analysis for category A with *diagnosed* Complex PTSD or other Complex PTSD indicators (>50% personality disorder).

In these seven studies, numbered 1–7 in tables and text (see also references: marked with *), four treatment conditions were distinguished: CBT, present centered therapy (PCT), treatment as usual during waiting list (TAU) and waiting list only. CBT was further subdivided into “including (prolonged) exposure” (PE) (imaginary or in vivo) and “including affect management” (AM) (skills training to improve affect regulation). Control conditions included TAU and WL.

### Calculation of study outcome measures

The following measures from the original studies were drawn upon for estimating PTSD effect sizes: (1) Clinician-Administered PTSD Scale (CAPS; Blake et al., 1995); (2) PTSD Symptom Scale—Self-Report (PTSD-SR; Foa, Cashman, Jaycox, & Perry, 1997); (3) Modified Posttraumatic Stress Disorder Symptom Scale—Self-Report (MPSS-SR) (Falsetti, Resnick, Resick, & Kilpatrick, 1993); and (4) Davidson Trauma Scale (Davidson et al., 1997). Two calculations were performed for each original study: (1) an assessment of effect sizes defined as the standardized pre–post score difference of the groups studied, and (2) a standardized score between conditions per study. For both calculations Cohen's d ([mean 1 − mean 2]/sd pre_pooled_) (Cohen, 1988) was used as the measure of the effect size using the following formula for the pooled standard deviation of the pretreatment scores: $s=\sqrt{\frac{(n_{1}-1)s_{1}^{2}+(n_{2}-1)s_{2}^{2}}{n_{1}+n_{2}}}$, resulting in an effect size also known as Glass's delta. We considered pre–post effect sizes (1) >0.2 small, >0.5 medium, and >0.8 large. For a direct comparison of two forms of treatment (2), we calculated the post/post effect sizes (d_post/post_), and corrected these effect sizes for group differences at the beginning of the study (d_pre/pre_) because such baseline differences may bias comparisons. Corrected effect sizes d_corr_ were obtained by computing d_corr_=d_post/post_−d_pre/pre_ (Becker, 1988; Morris, 2007; Seidler & Wagner, 2006). Between-condition effect sizes (2) are considered medium between 0.35 and 0.75.

Additionally, we present (1) percentage inclusion, conservatively defined as number of patients screened by researchers, even if prescreened during telephone interviews or by clinicians, (2) recovery rate, defined as percentage of patients who no longer met diagnostic criteria for PTSD, and (3) improvement rates using definitions for improvement as used by the authors for both completers’ and intention-to-treat analyses, with the aim to provide a comprehensive overview (Bradley et al., 2005). In case only completers’ (CPL) results were published, we assumed last observation carried forward (LOCF) imputation and used published drop-out rates to estimate non-reported intention-to-treat results. Similarly, if only intention-to-treat results were published, we estimated non-reported CPL results using drop-out rates and assuming LOCF.

Next, we computed a cumulative effect size of global PTSD symptoms across studies. First, a joint effect size was calculated for the three original studies using more than one PTSD measure. This joint effect size is equivalent to the arithmetical average of the global scale scores. Subsequently, the average global effect size was calculated from these primary-study effects, using fixed effect weights with Metan software (Borenstein, Hedges, Higgins, & Rothstein, 2009). Subsequently, we computed confidence intervals for both continuous (pre–post) as well as binary data (drop-out, recovery, and improvement rates). For confidence intervals the proportions of overlap were computed to establish if the pooled data of two groups of studies differed significantly (Cumming & Finch, 2005). Non-overlapping intervals have a corresponding *p*<0.01, and intervals overlapping no more than 0.5 have a corresponding *p*<0.05.

## Results

### Population characteristics of CA-related PTSD or Complex PTSD studies

Table 1 lists population characteristics of the seven included studies. The mean age of study populations ranged between 34 and 40 years, most populations, except two,^3,6^ were predominantly Caucasian, most patients were well educated and employed. Three studies^2–4^ reported advertisements as their method of recruitment and two studies only included referred patients.^1,7^

**Table 1:** Study characteristics of randomized controlled trials with CA-related (Complex) PTSD (A+B): *N*, population, recruitment, inclusion and exclusion criteria, assessment and severity of trauma and symptoms and previous treatments

							Assessment			Severity			
								
Author, year	*N*	Population	Recruitment	Inclusion criterion	Exclusion criteria	Inclusion rate^a^	Trauma	Axis I	Axis II	Trauma	Symptom	Comorbidity	Previous treatments
Zlotnick et al., 1997 (A)	48	All women Mean age 39 yrs 99% White 33% College degree 29% Low income	Referred	CSA-related Complex PTSD (DSM-IV)	Psychosis, substance abuse, DID	NR	CTQ	CAPS SIDES	NR	PTSD based on sexual abuse before age 17, above threshold CTQ 77% CSA by relative 35% parent 37% rape Mean age of onset 6.9 yrs; Mean 3.7 abusers	DTS 70 DES 22	100% Complex PTSD	50% previous hospitalization
Classen, Koopman, Nevillmanning, & Spiegel, 2001 (B)	55	All women Mean age ca. 38 yrs 64% White 44% College degree 80% Employed	Advertisement newspapers, flyers, radio, community agencies	CSA-related PTSD (DSM-IV)	Schizophrenia, dementia, delirium, amnesic/cognitive disorders, ritual abuse, current psychotherapy, alcohol/drug dependence, current suicidality (last month)	58%	SES	TSC-40	NR	At least two explicit memories of sexual abuse that involved genital contact; at least two sexual abuse events between 3–15 yrs of age; perpetrator at least 5 yrs older.	Only difference scores reported	NR	NR
Cloitre et al., 2002 (B)	58	All women Mean age 34 yrs 46% White 52% College degree 75% Employed or student	Self-referred advertisements	CA-related PTSD (DSM-IV)	BPD, Organic or psychotic mental disorder, substance dependence, eating disorder, dissociative disorder, bipolar disorder-I, suicide attempt or hospitalization during last 3 months	56%	CMIS SAAIVS	CAPS Blind for condition SCID-I	SCID-II	At least 1 clear memory - CSA: at least 1 time sexual contact before age 18 perpetrator at least 5 yrs older - CPA: parent/other adult in charge purposely hurt leaving e.g., bruises 48% CSA+CPA 39% CSA “only” 13% CPA “only”	CAPS 70 MPSS-SR 70 BDI 24	45% MDD 79% anxiety disorder 25% past substance abuse 16% past eating disorder	29% need of ER past year
Chard, 2005 (B)	71	All women Mean age ca. 33 yrs Mean education 14 yrs 81% White	Advertising, letters, presentations to local health professionals.	CSA-related PTSD (DSM-IV)	Suicidal intent, substance dependence, current trauma, medical disorders, unstable medication during last 3 months, substance abuse during last 3 months	81%	STI SAEQ	CAPS SCID-I	NR	At least 1 sexual incident as defined by state law, at least 1 memory Mean age of onset 6.4 yrs 57% >100 times, 63% >1 abuser, 84% by a relative	CAPS 67 BDI 24 DES 19	40% MDD	NR
McDonagh et al., 2005 (A)	74	All women Mean age ca. 40 yrs	NR	CSA-related Complex	NO personality disorder was an exclusion criteria. Medical disorders, (hypo)	30%	ELS	CAPS SCID-I	SCID-II	Sex contact with anyone at least 5 yrs older when under 16. PTSD	CAPS 70 BDI 18 DES 15	100% personality disorder	NR
		95% White 80% High school or higher 83% Employed	NR	PTSD (DSM-IV)	mania, schizophrenia, schizoaffective disorders, psychosis, DID, organic psychosis, severe, bipolar or psychotic depressive disorder, current alcohol/drug abuse last 3 months, active suicidality, history of 2 suicide attempts, abusive partner	30%	ELS	CAPS SCID-I	SCID-II	intrusion (partly) related. At least 1 clear detailed memory Much CSA+CPA and much adult abuse, majority involved penetration by a relative	DES 15	(10.8% BPD)	NR
Cloitre et al., 2010 (A)	104	All women Mean age ca. 36 yrs 35% white 88% high school 33% unemployed	NR	CA-related Complex PTSD (DSM-IV)	Substance dependence, psychotic symptoms, cognitive impairment, bipolar disorder, active suicidality requiring ER, current PTSD focused psychotherapy	34%	NR	CAPS Blind for condition	SCID-II	Primary diagnosis of CA-related PTSD by care taker/someone in authority before age 18 90% CSA 80% CPA Much additional adult abuse Mean 6.5 traumatic experiences	CAPS 64 BDI 21	90% Axis I diagnosis 50% personality disorder (24% BPD)	Mean 2 previous psychological treatments
Dorrepaal et al., 2012 (A)	71	All women Mean age 40 yrs Mean education 9.9 yrs 17% employed	Referred patients	CA-related PTSD and Complex PTSD (DSM-IV)	Long-lasting psychosis, DID, severe substance abuse interfering with compliance, antisocial personality disorder	79%	STI	CAPS SIDES blind	96% Axis I (55% MDD) 75% Axis II (52% BPD) 70% used psychotropic medication (47% SSRI/SNRI, 25% sedatives, >50% antipsychotics)	CA before age 16 as defined by STI 93% CSA >50% CSA+CPA >50% additional adult abuse	DTS 85 DES 25	96% Axis I (55% MDD) 75% Axis II (52% BPD) 70% used psychotropic medication (47% SSRI/SNRI, 25% sedatives, over 50% antipsychotics)	Mean 2 previous psychological treatments

BDI=Beck Depression Interview; BPD=borderline personality disorder; CAPS=Clinician-Administered PTSD Scale; CBT=cognitive behavioral therapy; CMIS= Childhood Maltreatment Interview Schedule; CA=child abuse; CPA=child physical abuse; CSA=child sexual abuse; CTQ= Childhood Trauma Questionnaire; DES=Dissociative Experiences Scale; DID=dissociative identity disorder; ELS=evaluation of lifetime stressors; ER=emergency room; MDD=major depressive disorder; MPSS-SR=Modified Posttraumatic Stress Disorder Symptom Scale—Self-Report; NR=not reported; SAAIVS= Sexual Assault and Additional Interpersonal Violence Schedule; SAEQ=Sexual Abuse Exposure Questionnaire; SCID-I/SCID-II=Structured Clinical Interview for DSM-IV—for Axis I/Axis II; SES=Sexual Experiences Survey; SIDES=Structured Interview for Disorders of Extreme Stress; SIDP-IV=Structured Interview for DSM-IV Personality Disorders; STI=Structured Trauma Interview; TSC-40=Trauma Symptom Checklist-40.

a Included patients/recruited patients×100%.

Common exclusion criteria were the presence of organic brain disorder, psychotic disorder, and substance abuse or dependence (Table 1). Suicidality was an exclusion criterion in five of seven studies.^2–6^ One^6^ excluded suicidality only if it required referral to a hospital. The two studies^1,7^ with populations diagnosed with Complex PTSD did not exclude suicidality. Dissociative (identity) disorder^1,3,5,7^ was excluded in four of seven studies. Other comorbid conditions like eating disorders,^3^ bipolar disorders,^3,5,6^ and severe depression,^5^ borderline^3^ or antisocial PD^7^ were sometimes excluded. Two studies^4,5^ excluded patients with ongoing abuse. Two^2,5^ studies used criteria involving clear memories of abuse under the age of 16 years by someone at least 5 years older.

Inclusion rates after screening were provided in six of seven studies^2–7^ and ranged between 30 and 81%, with a mean of 56% (Table 1). Two studies^5,6^ categorized as Complex PTSD populations showed low inclusion rates. In view of the low inclusion rate and exclusion criteria of two (A) studies,^5,6^ some caution regarding their generalizability to the Complex PTSD population is thus warranted.

Six studies diagnosed PTSD with the CAPS,^1,3–7^ and three studies reported blinded measurements.^3,6,7^ With regard to comorbidity, three studies^3,4,7^ reported on comorbid major depressive disorder (MDD) (40–55%) and two^6,7^ reported a mean of two or more other Axis I diagnoses. Four studies^3,4,5,6^ reported on depression severity using the Beck Depression Interview, with mean scores ranging from 18 to 25 (cutoff for moderate depression 16; severe 24). Dissociation was measured four times with the DES, with scores ranging from 15 to 25^1,4,5,7^ (the cutoff being 15–20). The four Complex PTSD (A) studies provided some information about personality pathology: 100% Complex PTSD as measured with the SIDES (including personality pathology) in two studies^1,7^ and two studies with at least 50% personality disorders^5,6^ (in study five this was estimated based on 15 of 29 patients meeting criteria for Borderline or Avoidant PD in the CBT condition). BPD comorbidity ranged from 11 to 53%.^5,6,7^

Only three studies reported on previous treatments.^1,3,7^ Participants’ trauma history was extensive throughout studies: many incidents were reported of multiple and severe types of CA, mainly by fathers/relatives, as well as a high adult abuse prevalence. Medication use was only reported once,^7^ showing that 70% of patients used psychiatric medication, including 20% antipsychotic medication.

### Treatment characteristics

The seven RCTs included a total of 17 conditions: 11 treatment conditions and six control conditions (four WL only, two TAU during WL [Table 2]). Two studies^2,5^ included two active treatments, and one^6^ compared three active treatment conditions.

**Table 2:** Treatment characteristics of randomized controlled trials with CA-related (Complex) PTSD (A+B)

1st Author, year	Active treatment	Format	Index trauma	Target symptoms	Psycho-education	CT/CR	Affect management	Exposure techniques	Social skills	Home work
Zlotnick et al., 1997 (A)	AM	15 weekly 90 min group sessions added to TAU	CSA	PTSD and affect dysregulation	+	±2 sessions	+	−	−	+
Classen et al., 2001 (B)	TFT+PFT	24 weekly 90 min group sessions	CSA	TFT: Work through, integrate. PFT: Modify maladaptive patterns	+	PFT+some basic assumptions	PFT: ±?	TFT: +?	Interpersonal learning?	?
Cloitre et al., 2002 (B)	STAIR+PE	8 weekly 60 min & 8 twice-a-week individual 90 min sessions	CA	PTSD and affect regulation	STAIR: +	+	STAIR: +	PE: +	STAIR: +	+
Chard, 2005 (B)	CPT-SA	17 weekly 90 min group sessions combined with 10 weekly 60 min individual sessions	CSA	PTSD, fear and attachment,	+	CPT-SA: +	_	WA: +	−	+
McDonagh et al., 2005 (A)	CBT PCT	7 weekly 120 min sessions followed by 7 individual 90 min sessions	CSA	CBT: Fear extinction and cognitive restructuring PCT: Change in traumagenic dynamics	+	CBT: + PCT: −	CBT: − PCT: Problem solving	CBT:+(PE) PCT: −	CBT: − PCT: Problem solving skills	+
Cloitre et al., 2010 (A)	STAIR/PE STAIR/Sup Sup/PE	16 weekly individual sessions	CSA	PTSD and affect dysregulation and interpersonal difficulties	PE: + STAIR: +	PE: + STAIR: +	PE: − STAIR: +	PE: + STAIR: −	PE: + STAIR: +	PE: + STAIR: +
Dorrepaal et al., 2012 (A)	CBT group	20 weekly 120 min group sessions added to TAU	CA	PTSD and Complex PTSD (Affect dysregulation, Dissociation, Self-esteem, Interpersonal difficulties, Somatization and Future meaning)	+	+	+	−	+	+

AM=affect management; C(S)A=child (sexual) abuse; CBT=cognitive behavioral therapy; CPT-SA=cognitive processing therapy for sexual abuse survivors; CR=cognitive restructuring; CT=cognitive therapy; PE=prolonged exposure; PFT=present focused therapy; STAIR=Skills Training in Affect and Interpersonal Regulation; Sup=supportive treatment; TFT=trauma focused therapy; WA=written accounts.

The total number of patients was 482, with 307 receiving active treatment, 119 WL only, and 56 TAU during WL. Six of seven studies^1,3–7^ provided data to calculate effect sizes, five completers’ ^1,3,4,5,7^ and three^5–7^ intention-to-treat data. These studies included at least one CBT condition, with eight CBT conditions in total (Table 2), and one included a PCT condition based on traumagenetic dynamics. These nine treatment conditions were manualized, all reporting psycho-education and homework as components of the treatment (Table 2). All CBT conditions included cognitive therapy and/or restructuring. Five treatment conditions^1,3,6^ ^(2x),7^ explicitly aimed at improvement of affect regulation and dedicated a substantial part of the treatment to AM skills training. Five conditions^2,3,5,6^ ^(2x)^ included exposure elements. Four conditions^3,^ ^6^ ^(2x),7^ addressed interpersonal functioning in explicit interpersonal skills training. Two conditions^2^ consisted of group treatment only, in three conditions group treatment was combined with individual care, once manualized,^4^ two times with unstructured TAU.^1,7^ Six conditions consisted of individual treatment only.^3,5,6^ Length of treatment ranged from 12 to 24 weeks or 14 to 27 sessions with a duration of 60–120 min each.

### Drop-out

The mean overall drop-out rate was 22%, in active treatments 25% and in control conditions 16% (Table 3 per study and Table 4 aggregated data). CBT had higher drop-out rates as compared to PCT. Active CBT conditions^4–6^ including some form of exposure without preceding AM showed a mean drop-out rate of 32%, while the three conditions^1,6,7^ without exposure showed a mean drop-out rate of 24%. In the two studies with direct comparisons between active treatment conditions,^5,6^ exposure conditions had a mean drop-out rate of 40% as compared to a mean drop-out of 18% in the no-exposure active treatments conditions. Three studies^1,4,5^ reported characteristics of drop-out patients, including higher PTSD^1,4^ and dissociation levels,^1^ more severe trauma,^5^ anxiety,^5^ and depression. One study^5^ also reported 100% drop-out in the exposure condition for BPD as compared to 0% in the PCT, while another^7^ found lower drop-out rates for BPD in the AM condition. *Within the Complex PTSD studies* (A) the comparison between different types of CBT showed lower drop-out in AM only and AM combined with exposure as compared to exposure only (Table 5).

**Table 3:** Drop-out rates, pretreatment and posttreatment scores (mean, SD), and effect sizes of pretreatment versus posttreatment and treatment versus waiting list or other treatment, recovery and improvement rates on PTSD symptom severity (completers and intention-to-treat) per included study

						Posttreatment score				Effect size				Post			
								
				*MEASURE*/Prescore		Completer		ITT		pre vs post (Cohen's d)		treatment vs. control (Cohen's d corrected)		Recovery rate		Improvement rate	
										
	Treatment or WL	N (N completer)	Drop-out	M	SD	M	SD	M	SD	Completer	ITT	Completer	ITT	Completer	ITT	Completer	ITT
Zlotnick et al., 1997 (A)	AM TAU+WL	24 (16) 24 (17)	33% 29%	*DTS* 66.9 74.7	22.00 25.83	45.76 73.06	34.12 29.86	52.2^a^ 73.5		0.91 0.07	0.63^a^ 0.05	0.84	0.58^a^	87% 41%	61%^a^ 30%	–	–
Classen et al., 2001 (B)	TFT+PFT WL only	14+7 34	NR	*TSC-40* g – –	– –	– –	– –	– –	– –	8.1 3.8	17.0 14.1	– –	– –	– –	– –	– –	– –
Cloitre et al., 2002 (B)	STAIR+PE WL only STAIR+PE WL only	31 (22) 27 (24)	29% 11%	*MPSS-SR* 69 73 *CAPS* 69 69	16.6 18.6 16.3 16.6	29 58 31 62	27.6 28.6 25.2 22.7	40.6^a^ 59.7 42.0 62.7		2.31 0.87 2.36 0.43	1.64^a^ 0.77 1.68 0.39	1.45 1.93	0.87^a^ 1.29	77% 25%	55%^a^ 22%	46% 4%	33%^a^ 4%
Chard, 2005 (B)	CPT-SA WL (MA) CPT-SA WL (MA)	36 (30) 35 (28)	17% 20%	*CAPS* 65.5 68.3 *MPSS-SR* 57.6 57.5	26.4 23.7 22.9 24.7	9.00 63.0 7.54 57.7	11.0 30.7 9.51 27.5	18.4^a^ 64.0 15.9 57.7		2.29 0.22 2.14 −0.01	1.91^a^ 0.17 1.78 −0.01	2.07 2.15	1.73^a^ 1.79	93% 26%	78%^a^ 21%	79% 4%	66%^a^ 3%
McDonagh et al., 2005 (A)	CBT PCT WL only	29 (17) 22 (20) 23 (20)	41% 9% 13%	*CAPS* 67.1 67.5 70.0	18.4 15.1 16.9	38.5 44.9 62.5	27.7 22.1 17.0	53.1 47.2 65.5	28.8 22.4 18.6	1.75 1.39 0.44	1.03 1.26 0.40	1.29 0.93 0.37^c^	0.63 0.86 −0.23^c^	47% 35% 20%	28% 32% 17%	–	–
Cloitre et al., 2010 (A)	STAIR/PE STAIR/Sup Sup/PE STAIR/PE STAIR/Sup Sup/PE	33 (28) 38 (28) 33 (20)	15% 26% 39%	*CAPS* 63.1 64.3 64.5 *PTSD-SR* 36.7 39.9 38.2	18.3 21.2 15.9 12.9 12.7 11.1	*27*.3^b^ 20.9 23.6 9.95 5.43 6.52		32.7 32.3 39.7 14.0 14.5 19.0	19.4 23.0 18.3 11.5 12.8 9.83	1.94^b^ 2.35 2.22 2.21 2.85 2.84	1.65 1.73 1.34 1.87 2.10 1.59	−0.28^d^ 0.14^e^ −0.41^f^ −0.64^d^ 0.23^e^ −0.41^f^	0.30^d^ 0.39^e^ −0.09^f^ −0.22^d^ 0.51^e^ 0.29^f^	72%^b^ 64% 55%	61% 47% 33%	32%^b^ 33% 10%	27% 24% 6%
Dorrepaal et al., 2012 (A)	CBT group TAU+WL	38 (31) 33 (29)	18% 12%	DTS crsf 91.4 80.5	21.8 23.1	66.7 65.5	29.4 30.3	69.6 66.5	27.4 29.8	1.12 0.68	0.99 0.63	0.44	0.35	–	–	55% 24%	45% 21%

AM=affect management; CAPS=Clinician-Administered PTSD Scale; CBT=cognitive behavioral therapy; CPT-SA=cognitive processing therapy for sexual abuse survivors; DTS=Davidson Trauma Scale; ITT=intention-to-treat; M=mean; MA=minimal attention; MPSS-SR=Modified Posttraumatic Stress Disorder Symptom Scale—Self-Report; NR=not reported; PTSD-SR=PTSD Self-Report; PE=prolonged exposure; PFT=present focused therapy; SD=standard deviation; STAIR= Skills Training in Affect and Interpersonal Regulation; TAU=treatment as usual; TSC-40=Trauma Symptom Checklist-40; TFT=trauma focused therapy; WL=waiting list.

a Mean ITT is estimated from mean completers, N ITT and N completers, assuming non-completers did not change;

b mean completers is estimated from mean ITT, N completers and N ITT, assuming non-completers did not change;

c CBT vs. PCT;

d STAIR/PE vs. Sup/PE;

e STAIR/Sup vs. Sup/PE;

f STAIR/PE vs. STAIR/Sup;

g change scores as reported in article, unknown if standardized.

**Table 4:** Aggregated drop-out, prescores and postscores, and effect sizes for PTSD symptom changes, and recovery and improvement rate across CA-related PTSD studies by type of treatment

						PTSD change score					Post					
							
			PTSD score			Pre versus post		Treatment versus control								
									
			Pre*	Post*		Effect size (d)		Effect size (d) post–post corrected			Recovery rate^8^			Improvement rate^9^		
							
Treatment type	*N*	Drop-out (%)		Completer	ITT	Completer	ITT	*N*	Completer	ITT	*N*	Completer (%)	ITT (%)	*N*	Completer (%)	ITT (%)
All active conditions together (A+B)	9	25^a^	68	34	42	1.7^c^	1.3^f^	6	1.2	0.9	8	68^g^	50^i^	6	45^j^	34^l^
CBT^1^,^3^,^4^,^5^,^6^,^7^	8	26^b^	68	33	42	1.7	1.3	5	1.3	0.8	7	72^h^	52	6	45	34
PE no AM^4^,^5^,^6^	3	32	64	23	36	2.1^d^	1.4	2	1.7^n^	1.2^o^	3	70	48	2	52	38
AM no PE^1^,^6^,^7^	3	24	74	44	51	1.4^d^,^e^	1.1	2	0.6^n^,^p^	0.4^o^	2	73	52	2	44	34
AM & PE^3^,^6^	2	22	66	29	37	2.1^e^	1.7	1	1.6^p^	1.1	2	74	58	2	38	30
PCT^5^	1	9^b^	68	45	47	1.4	1.3	1	0.9	0.9	1	35^h^	32	0		
Control conditions^1^,^3^,^4^,^5^,^7^	5	16^a^	72	64	65	0.4^c^	0.3^f^	0			4	27^g^	22^i^	3	11^j^	9^l^
WL only^3^,^4^,^5^	3	15	68	61	62	0.4	0.3	0			3	24	20	2	4^k^	3^m^
TAU during WL^1^,^7^	2	18	78	69	70	0.4	0.4	0			1	41	30	1	24^k^	21^m^

AM=affect management; CA=child abuse; CBT=cognitive behavioral therapy; ITT=intention-to-treat; PCT=present centered treatment; PE=prolonged exposure; TAU=treatment as usual; WL=waiting list.

* Without Cloitre et al., 2010 due to incomparability of score ranges.

1 Zlotnick et al., 1997;

2 Classen et al., 2001;

3 Cloitre et al., 2002;

4 Chard, 2005;

5 McDonagh et al., 2005;

6 Cloitre et al., 2010;

7 Dorrepaal et al., 2012;

8 Percentage decrease of PTSD diagnoses as reported by author;

9 Percentage of patients that improved as defined by author.

Pre–post effect sizes, recovery and improvement rates were tested: active vs. control; CBT vs. PCT; the three subtypes of CBT amongst each other; and the two types of control conditions:

a,b,d,e,k,m,o Differences between groups of studies with the same superscript are significant at *p*<0.05 as a result of modest overlap of confidence intervals.

c,f,g,h,i,j,l,p,n Differences between groups of studies with the same superscript are significant at *p*<0.01 as a result of non-overlap of confidence intervals.

**Table 5:** Aggregated drop-out, prescores and postscores, and effect sizes for PTSD symptom changes, and recovery and improvement rate across CA-related Complex PTSD studies by type of study population

						PTSD change score					Post					
							
			PTSD score			Pre versus post		Treatment versus control								
											
			Pre*	Post*		Effect size (d)		Effect size (d) post–post corrected			Recovery rate^8^			Improvement rate^9^		
							
Study population	*N*	Drop-out (%)		Completer	ITT	Completer	ITT	*N*	Completer	ITT	*N*	Completer (%)	ITT (%)	*N*	Completer (%)	ITT (%)
CA-related Complex PTSD (A)^1^,^5^ ^(2x),^^6^ ^(3x),^^7^	7	26	69	38	46	1.6^d^,^e^	1.2^f^,^g^	4	0.8^t^	0.6^u^	6	60^h^,^i^	44^k^,^l^	4	35^o^	26^q^
CA-related PTSD (B)^3^,^4^	2	22	65	19	29	2.3^d^	1.8^f^	2	1.9^t^	1.4^u^	2	87^h^	67^k^	2	65^o^	51^q^
Within CA-related Complex PTSD (A):																
CBT	6	28^a^	70	37	46	1.6	1.2	3	0.7	0.5	5	65^j^	46	4	35	26
With PE no AM^5^,^6^	2	40^b^,^c^	66	31	45	2.0	1.2	1	1.2	0.6	2	51	31^m^,^n^	1	10^p^	6^r^,^s^
With AM no PE^1^,^6^,^7^	3	24^b^	74	44	51	1.4	1.1	2	0.6	0.4	2	73	52^m^	2	44^p^	34^r^
With AM & PE^6^	1	15^c^	63	27	33	1.9	1.6	0			1	71	61^n^	1	32	27^s^
PCT^5^	1	9^a^	68	45	47	1.4	1.3	1	0.9	0.9	1	35^j^	32	0		
Control conditions^1^,^5^,^7^	3	16	75	67	68	0.4^e^	0.4^g^				2	30^i^	24^l^	1	24	21
WL only^5^	1	13	70	62	65	0.4	0.4				1	20	17	0		
TAU during WL^1^,^7^	2	18	78	69	70	0.4	0.4				1	41	30	1	24	21

AM=affect management; CA=child abuse; CBT=cognitive behavioral therapy; ITT=intention-to-treat; PCT=present centered treatment; PE=prolonged exposure; TAU=treatment as usual; WL=waiting list.

* Without Cloitre et al., 2010 due to incomparability of score ranges.

1 Zlotnick et al., 1997;

2 Classen et al., 2001;

3 Cloitre et al., 2002;

4 Chard, 2005;

5 McDonagh et al., 2005;

6 Cloitre et al., 2010;

7 Dorrepaal et al., 2012;

8 Percentage decrease of PTSD diagnoses as reported by author;

9 Percentage of patients that improved as defined by author.

Pre–post effect sizes, recovery and improvement rates were tested: CA-related Complex PTSD (A) vs. CA-related PTSD (B); active vs. control; CBT vs. PCT and the three subtypes of CBT amongst each other, and the two types of control conditions:

a,b,c,d,f,j,l,m,s Differences between groups of studies with the same superscript are significant at *p*<0.05 as a result of modest overlap of confidence intervals.

e,g,h,i,k,n,o,p,q,r,t,u Difference between groups of studies with the same superscript are significant at *p*<0.01 as a result of non-overlap of confidence intervals.

In sum, PCT showed lowest drop-out rates. Within CBT treatments, drop-out rates for exposure were high compared to AM, significantly so in Complex PTSD studies.

### Effect sizes

Tables 3 and 4 indicate that active treatments resulted in substantial improvement from pretreatment to posttreatment in this patient population, with effect sizes ranging from 0.6 to 2.8 (Table 3) with a mean of 1.7 for completers and 1.3 for intention-to-treat (Table 4). The effect sizes of control conditions ranged from no effect to medium effect sizes with a small mean effect size of 0.4 in completers, and 0.3 in intention-to-treat (in WL only^3–6^ as well as TAU during WL^1,7^ conditions). The effect sizes of active treatments versus control (WL only plus TAU during WL) comparisons ranged from 0.4 to 2.2 (Table 3), with large mean effects of 1.2 in completers and 0.9 in intention-to-treat (Table 4).

In Table 4 we also aggregated data for CA-related PTSD (A and B) for each active condition, showing large effect sizes pre post for all types of active treatment. The 95% confidence interval for treatments including exposure only (EXP no AM) in contrast with AM only treatments (AM no EXP) indicated more favorable results for exposure in completers, in line with treatment versus control effect sizes. Additionally, we observed small to medium effect sizes (ranging from 0.09 to 0.51) in direct comparisons between active treatments within studies^5,6^ (Table 3): both in pre–post as well as treatment versus other treatment effect sizes, relatively favorable results for exposure only were found in completers’ analyses, whereas in intention-to-treat analyses relatively unfavorable results for exposure only were found.

Comparing pre–post effect sizes in CA-related *Complex* PTSD studies^1,5–7^ (A) versus CA-related non-Complex PTSD studies^3,4^ (B) revealed less treatment gain for the complex population (Table 5) in completers’ analyses, as confirmed in intention-to-treat analyses. We additionally compared treatment gains per type of treatment within the Complex PTSD studies only (category A) (Table 5) and failed to observe evidence for differential pre–post effect sizes between active treatments. The relatively modest results in the more complex populations were also evident within the (A) category. The two studies^1,7^ that included Complex PTSD *diagnosed* patients showed lower effect sizes (Table 3) compared to the studies^5,6^ that included a PTSD diagnosed population with a minimal 50% personality disorder comorbidity as indicator of complexity, possibly also including less complex patients.

### Recovery rates

Five studies^1,3–6^ reported outcome in terms of recovery rate. In eight active conditions a mean recovery rate of 50% was found, comparing favorably with a recovery rate of 22% in the control conditions (Table 4) in intention-to-treat analysis, also found in completers’ analysis. Table 4 shows a higher recovery rate for CBT as compared to PCT in completers, but not in intention-to-treat. No difference was observed in the direct comparison^5^ (Table 3). In contrast to effect size comparisons, no evidence for differential recovery rates between types of CBT was found.

The Complex PTSD studies (A) showed a lower recovery rate as compared to the PTSD studies (B) in intention-to-treat analysis, which also was seen in completers’ analysis, in line with differential effect sizes (Table 5). Within Complex PTSD studies, we now found higher recovery rates for AM only versus exposure only in intention-to-treat analysis (Table 5). Similar findings were obtained for combined exposure and AM versus exposure only.

Taken together, overall modest recovery rates were observed, favoring CBT over PCT in completers’ analysis not in intention-to-treat. Again, in the Complex PTSD studies recovery rates were less favorable compared to the PTSD studies (B). Recovery rates for AM (only or in combination with exposure) exceeded those for exposure only in Complex PTSD studies.

### Improvement rates

Active treatments resulted in mean improvement rates of 45% in completers and 34% in intention-to-treat (Table 4). Although this contrasts favorably with 11 and 9%, respectively, in control conditions, a majority of patients failed to reach this criterion. No differential improvement rates between CBT and PCT or types of CBT were found. Improvement rates for TAU during WL were more favorable than for WL only.

Again, Complex PTSD patients (A) showed unfavorable results in terms of improvement rate: 35% versus 65% in DSM-defined PTSD patients (B) in completers, as well as in intention-to-treat analysis (Table 5). Between types of treatment within the Complex PTSD (A) studies, exposure only showed lower improvement rates (Table 5) as compared to AM only in both intention-to-treat and completers’ analysis. Lower improvement rates for exposure only were also found in direct comparisons^5,6^ listed in Table 3.

In sum, most patients failed to reach criteria for significant improvement. In the Complex PTSD studies improvement rates were even lower, especially for exposure as compared to AM, in line with results for recovery rates.

### Pretreatment and posttreatment symptom level

The mean pretreatment score on the CAPS was around 70 (range 64–86), indicative for severe PTSD. Posttreatment, the mean score for completers of active treatment conditions was 34 (range 9–67) (Tables 3 and 4). In the PTSD (B) studies, active conditions resulted in a comparatively favorable mean post score of 19, whereas the Complex PTSD (A) studies revealed a mean post score of 38 for completers (Table 5), which is approximately the cutoff score for a PTSD diagnosis, while in intention-to-treat participants these scores were even higher.

These findings therefore indicate that predominantly CBT treatments are effective in reducing PTSD in the CA-related PTSD population, but fall short in meeting the needs of the Complex PTSD population.

### Complex PTSD outcome measures

Data on Complex PTSD domains could not be aggregated due to a limited number of measurements and their heterogeneity. Outcome variables that were measured in more than one study included dissociation, interpersonal problems, depressive symptoms, and anger. Results were however mixed, as some improved similarly to PTSD scores, whereas others did not, so that definitive conclusions cannot yet be drawn.

## Discussion

Our research question was: What empirical evidence is available to effectively treat the subgroup of CA-related Complex PTSD patients and to guide our choice of optimal treatment? Little guidance was found in the currently available literature, because to our knowledge no review on CA-related PTSD or Complex PTSD has been published to date, and reviews concerning CA or PTSD do not provide clear evidence for optimal treatment of these complex patients.

We identified 24 RCTs addressing a combination of PTSD and CA, which were however highly variable with regard to study population, index trauma, treatment target, and outcome measures. We sorted these RCTs into five groups of studies (A–E), depending on the target populations: consisting of (four) combinations of PTSD or mainly PTSD with CA or mainly CA, combined with a (fifth) group of Complex PTSD and CA. For the purpose of this review, we concentrated on the studies assigned to the categories A (Complex PTSD and CA) and B (PTSD and CA). In the category A and B studies, all patients were diagnosed with PTSD, all suffered from CA as the index trauma and the treatment target was PTSD or Complex PTSD. Category A likely included a *Complex PTSD* population, which was therefore analyzed separately. Studies assigned to category C focused on PTSD patients, which only partly suffered from a CA history and CA was not the index trauma in the majority of the patients. In category D, the patients had a CA history, and CA was the index trauma; however, only parts of these patients were diagnosed with PTSD and this subgroup was not analyzed separately. Category E pertained to studies of patients only partly suffering from CA and partly from PTSD.

The patients of the category A and B studies predominantly consisted of Caucasian patients, who were mostly well educated, employed, and severely traumatized. Suicidal and dissociative (identity) disorder patients were frequently excluded. The included patients were recruited mostly by advertisements. Information regarding previous treatments including hospitalizations and medication use was lacking in most studies, limiting generalizability to “real life” populations.

Results of the meta-analysis showed that these patients improved substantially following different types of treatments targeting CA-related PTSD or Complex PTSD, as indicated by large effect sizes. Patients in active treatment conditions, predominantly cognitive behavioral treatments, showed superior outcomes in recovery and improvement rates compared to control conditions. However, post treatment symptom scores were still substantial: just less than half of patients no longer met criteria for a PTSD diagnosis and only a minority showed clinically relevant improvement. CBT and PCT were generally equally effective, but differed in drop-out rate favoring PCT, and recovery rate favoring CBT in completers’ analysis. CBT treatments ranged from AM to exposure, always including psycho-education and cognitive therapy. Between CBT types, exposure resulted in greater effect sizes as compared to AM, although recovery and improvement rates were similar.

When comparing CBT treatment outcome after 12–24 weeks for the most Complex PTSD populations (assigned to category A) with PTSD populations (assigned to category B), we found that the Complex PTSD population benefitted less from treatment as compared to DSM-defined PTSD. Moreover, no differential treatment effect sizes were observed in Complex PTSD. In contrast, for Complex PTSD patients, more favorable drop-out, recovery, and improvement rates for AM were observed compared with exposure treatment.

These findings indicate that despite the large effect sizes, which are in line with earlier PTSD reviews, the presence of substantial symptoms post treatment together with low to moderate improvement and recovery rates imply a clear need for better treatments. This is even more the case for the Complex PTSD population in which the results are less favorable as compared to the PTSD population. Even within the four Complex PTSD studies effect sizes are highest in the studies^5,6^ with more exclusion criteria and lower inclusion rates. This is in agreement with the finding in the meta-analysis of Bradley et al. (2005) showing that higher effect sizes are related to more exclusion criteria. This limits generalizability to the most impaired Complex PTSD patients and stresses the importance of developing and studying treatment improvements for this group with high rates of comorbidity, suicidality, unemployment, and utilizing costly intensive treatment including medication and hospitalization.

Additionally, a considerable proportion of patients drop-out of treatment, without relief of their disturbing symptoms, whereas the remaining patients generally achieve large effects in completers’ analyses. Our findings indicate the relevance of comparing results between (sub) populations, which may be illustrated by a difference in drop-out between exposure and AM in the Complex PTSD population, but not in the PTSD populations. Probably, patients who are able to tolerate exposure are likely to complete their treatment successfully. The differential drop-out rates between PCT (9%) and CBT (26%) as well as between exposure and other treatments stress the importance of additional intention-to-treat analyses to obtain more balanced results. These findings concur with a previous report showing higher drop-out during exposure therapy in more complex patients (with comorbid personality disorder) (McDonagh et al., 2005), whereas within a Complex PTSD population the least complex patients (without comorbid BPD) drop-out more frequently during AM (Dorrepaal et al., 2012). In the literature it has been noted that drop-out risk may be highest in the first exposure sessions (McDonagh et al., 2005) and may decrease after improvement of negative mood regulation or achieving a more robust working alliance (Cloitre, Koenen, Cohen, & Han, 2002).

Systematic inventories among experts recommend an initial focus on “stabilization” in Complex PTSD patients as well as dissociative disorders with an inability to tolerate strong affects before exposure (Baars et al., 2011; Brands et al., 2012; Cloitre et al., 2011). Thus, regular exposure treatments may be unsuitable for Complex PTSD patients in the first phase of their treatment. More psycho-educational or stabilizing treatments targeting affect dysregulation, irrational beliefs, and/or lack of social and self-soothing skills may prepare patients to subsequent treatments such as exposure (e.g., Harned, Jackson, Comtois, & Linehan, 2010), or directly reduce PTSD symptoms in other cases (e.g., Zlotnick et al., 1997).

It is unknown if treatment outcome can be improved by varying treatment type and duration, since most treatments studied to date are 12–24 week CBT treatments. An integrated approach focusing not only on PTSD outcome but also on (complex) associated features is therefore mandatory. For example, a treatment schedule starting with AM was shown to be both tolerable as well as effective (Cloitre et al., 2002, 2010), although not tested yet in a fully diagnosed Complex PTSD population, or analyzed separately for the PD subgroup.

### Strengths and limitations

In contrast to most PTSD meta-analyses, which are only based on the results of completer analyses, we additionally presented aggregated intention-to-treat data, and when necessary estimated these based on drop-out rates and completers’ results. This is of relevance since drop-out rates differed between treatments. Additionally, we calculated multimodal outcome measures, presenting not only effect sizes but also recovery and improvement rates, inclusion rates and postscores, resulting in a more comprehensive overview. Moreover, we attempted to focus on well-circumscribed populations, thereby avoiding conclusions that would be over-generalizing and hence unspecific. However, even within the Complex PTSD study populations some characteristics like inclusion rates and exclusion criteria indicated different levels of complexity/severity.

From a methodological viewpoint, in performing the meta-analysis, the relatively low number of studies precluded rigorous testing and adjustment for homogeneity (although the random effects model gave very similar results compared to the presented results from the fixed effects model) and also impeded proper assessment of funnel plots in order to evaluate publication bias.

Due to our stringent inclusion criteria the number of identified studies was modest, limiting the power to identify differential treatment (e.g., length of treatment) or population effects. Additionally, different numbers of studies could be included per outcome, for example, effect size or recovery rate, limiting their interpretation. Moreover, we did not analyze follow-up data, which might provide important additional information, as for instance in the Cloitre et al. (2010) study most differential results were not evident before follow-up. Follow-up data did not allow meaningful aggregation: Two studies^1,7^ did not report follow-up data; two more studies^2,4^ did not report means and SDs needed to aggregate on follow-up; also generally no follow-up information of the control condition^3,5,6^ was given. Notwithstanding these limitations, findings indicate that gains are at least sustained and sometimes improved over follow-up, specifically concerning PTSD symptoms.^3–6^ We were only able to analyze treatment outcome in terms of PTSD symptom severity, which is likely to introduce a bias with regard to patients’ perceived needs and treatment effects. For example, a meta-analysis on CA studies found CBT treatments superior in terms of PTSD outcome, but not on externalizing problems (Taylor & Harvey, 2010). Moreover, the interpretation of the effects of Complex PTSD versus AM on treatment results may be confounded, since AM was used in Complex PTSD populations only. Lastly, we defined Complex PTSD study populations arbitrarily as Complex PTSD diagnosed or PTSD with at least 50% PD comorbidity, so the possibility that patients in a study population meeting the latter criteria did not all have Complex PTSD cannot be ruled out. Given the low inclusion rate, the presence of exclusion criteria like suicidality and low inclusion rate in these two Complex PTSD studies (Cloitre et al., 2010; McDonagh et al., 2005), and the fact that most patients were self-referred, Caucasian, well educated, and employed (except seven) indeed warrants some caution regarding the generalizability of the results, especially for exposure, to the most Complex PTSD population.

### Future research

Given the paucity of studies in CA-related Complex PTSD populations to date, additional trials are needed to explicitly address these patients, with careful assessments and minimal exclusion criteria. The main challenge is whether more favorable effects and lower drop-out rates can be achieved using established approaches for routinely included study populations, such as C(P)T vs. exposure vs. EMDR, comparing these to, e.g., personality disorder treatment programs. To investigate possible treatment×population interactions, a study comparing treatments having the same number of sessions within a Complex PTSD diagnosed population is warranted. This could also add to the results of Cloitre et al. (2010) who compared eight sessions of AM with eight sessions of exposure with 16 sessions of both of these modalities successively. It remains unclear whether extending the eight session stabilizing AM condition to 16 sessions would result in similar results as compared to the 16 session combined treatment or 16 sessions exposure only.

As noted earlier, to enhance generalizability of results it is important to broadly include referred populations with minimal exclusion criteria like suicidal behavior, dissociation or substance abuse, which are characteristic for the Complex PTSD population. In addition, personality disordered, non-Caucasian, lower educated, unemployed, medicated, and previously treated patients should be included and these characteristics should be reported and their relevance analyzed. This important knowledge gap as identified in this review corroborates earlier publications (Bradley et al., 2005; Cloitre, 2009, 2011). Axis II disorder assessment and separate analyses of patients with and without Axis II diagnoses are likewise needed to address questions regarding generalization of effects to more Complex PTSD populations. Moreover, differential drop-out and analysis on both completers as well as intention-to-treat are warranted to obtain a balanced overview. Finally, uniform measures for Complex PTSD symptoms are needed to allow comparisons between studies.

Concluding, the results of this review suggest that a variety of treatments may be effective for CA-related PTSD, but they may not be sufficient enough to obtain satisfactory end states in the more Complex PTSD populations. Therefore, it is important firstly to be able to differentiate properly between DSM-defined and Complex PTSD populations, and secondly to compare the effect of different combinations and sequences of a variety of treatment modalities in well-established Complex PTSD populations.

## Supplementary Material

Evidence-based treatment for adult women with child abuse-related Complex PTSD: a quantitative review

Evidence-based treatment for adult women with child abuse-related Complex PTSD: a quantitative review
