# Supplementary material for: Evidence-based treatment for adult women with child abuse-related Complex PTSD: a quantitative review
Source: Eur J Psychotraumatol. 2014 Oct 14;5:10.3402/ejpt.v5.23613. doi: 10.3402/ejpt.v5.23613 (PMC4199330; doi:10.3402/ejpt.v5.23613)
Supplement: Evidence-based treatment for adult women with child abuse-related Complex PTSD: a quantitative review [file EJPT-5-23613-s001.pdf]

Titel: Evidence-based behandeling voor volwassen vrouwen met Complexe PTSS na vroegkinderlijk trauma: een meta-analyse

Ethy Dorrepaal, Kathleen Thomaes, Adriaan W. Hoogendoorn, Dick J. Veltman, Nel Draijer and Anton J. L. M. van Balkom

Samenvatting

Inleiding: Uit wetenschappelijk onderzoek is bekend welke behandelingen effectief zijn voor een posttraumatische stress stoornis (PTSS), maar het is niet duidelijk of deze behandelingen ook effectief zijn voor Complexe PTSS na vroegkinderlijk trauma.
Methode: We deden een literatuursearch naar effectstudies over behandelingen die specifiek gericht zijn op PTSS na vroegkinderlijk trauma en voor Complexe PTSS. Er werd een meta-analyse gemaakt van deze studies met verschillende variabelen, zoals effect size, drop-out, en percentage herstelde en verbeterde patiënten (remissie resp. responders).
Resultaten: Slechts 6 studies met een of meer cognitieve gedragstherapie (CGT) condities en een met een Present Centered Therapie conditie voldeden aan de criteria voor de meta-analyse. De resultaten toonden dat patiënten met PTSS na vroegkinderlijk trauma duidelijk verbeterden (grote effect sizes) maar met matige herstel- en verbeterpercentages. Behandelingen met een exposure component toonden grotere effect sizes bij PTSS patiënten, vooral in de "completer" analyse, maar resulteerden niet in hogere herstel- en verbeterpercentages. Complexe PTSS patiënten reageerden minder goed op behandeling. Er was geen superieur effect van behandeling met een exposure elemant bij Complexe PTSS patiënten. In de Complexe PTSS groep, had emotieregulatie training gunstigere herstel- en verbeterpercentages en minder drop-out dan exposure, vooral in "intention-to-treat" analyse.
Conclusie: Op basis van deze meta-analyse met helaas nog weinig studies, kunnen we concluderen, dat voornamelijk CGT behandelingen bewezen effectief zijn bij PTSS na vroegkinderlijk trauma. Echter, in de Complexe PTSS patiënten wordt daarmee niet voldoende resultaat bereikt en lijkt emotieregulatie training beter resultaten op te leveren dan exposure. Toekomstig onderzoek zou zich moeten richten op directe vergelijkingen tussen de verschillende vormen van behandeling voor Complexe PTSS patiënten, om de generaliseerbaarheid van de resultaten te verbeteren.

Trefwoorden: Review, Meta-analyse, PTSS, Posttraumatische stressstoornis, Psychotherapie, cCognitieve gedragstherapie, Kindermishandeling, Kindermisbruik, Complexe PTSS.

Citation: European Journal of Psychotraumatology 2014, 5: 23613 - http://dx.doi.org/10.3402/ejpt.v5.23613
