# Supplementary material for: Evidence-based treatment for adult women with child abuse-related Complex PTSD: a quantitative review
Source: Eur J Psychotraumatol. 2014 Oct 14;5:10.3402/ejpt.v5.23613. doi: 10.3402/ejpt.v5.23613 (PMC4199330; doi:10.3402/ejpt.v5.23613)
Supplement: Evidence-based treatment for adult women with child abuse-related Complex PTSD: a quantitative review [file EJPT-5-23613-s002.pdf]

Un traitement fondé sur les faits pour des femmes adultes présentant un état de stress post-traumatique lié à une maltraitance infantile : une revue quantitative

Kathleen Thomaes, Ethy Dorrepaal, Adriaan W Hoogendoorn, Dick J Veltman, Nel Draijer, Anton J van Balkom

Introduction : les traitements efficaces de première intention pour l'état de stress post-traumatique (ESPT) sont bien établis, mais leur potentiel de généralisation à l'ESPT Complexe lié à la maltraitance infantile (MI) reste méconnu.
Méthode : une revue quantitative de la littérature a été réalisée, identifiant ainsi sept études, incluant des traitements spécifiques qui ciblent l'ESPT lié à la MI ou l'ESPT Complexe. Ces études méta-analysées incluaient des variables telles que la taille d'effet, les abandons, le rétablissement et les taux d'amélioration.
Résultats : seulement 6 études avec une ou plusieurs thérapies cognitivo-comportementales (TCC) et une étude incluant une thérapie « centrée sur le présent » ont pu être méta-analysées. Les résultats indiquent que les patients atteints d'ESPT lié à une MI bénéficient d'importantes tailles d'effet et de modestes rétablissements et taux d'amélioration. Les traitements incluant des techniques d'exposition ont présenté de plus grandes tailles d'effet, particulièrement chez les sujets ayant suivi le traitement complet, bien qu'aucun résultat différentiel n'ait été trouvé dans le rétablissement ou les taux d'amélioration. Cependant, les résultats dans le sous-groupe d'études sur l'ESPT Complexe lié à une MI ont été moins favorables. Dans le sous-groupe sur l'ESPT Complexe, aucune taille d'effet supérieure n'a été trouvée concernant les techniques d'exposition, et les techniques de gestion de l'affect ont résulté en de plus favorables rétablissements et taux d'amélioration et en moins d'abandons comparé aux techniques d'exposition, particulièrement dans les analyses avec « intention de traitement (ITT). »
Conclusions : des preuves limitées suggèrent que l'efficacité des TCC est prédominante, bien qu'elle ne suffise pas à atteindre des états finaux satisfaisants, spécialement dans les populations atteintes d'ESPT Complexe. De plus, nous proposons que les futures recherches devraient se focaliser sur la comparaison direct entre les types de traitement pour les patients atteints d'ESPT Complexe, augmentant ainsi le potentiel de généralisation des résultats.

Keywords: Revue ; Méta-analyse ; ESPT ; État de Stress Post-Traumatique ; Psychothérapie ; Thérapie cognitivo-comportementale ; Traitement cognitivo-comportemental ; Maltraitance infantile ; Survivants adultes de maltraitance infantile

Name of translator: Marc Legrand

Citation: European Journal of Psychotraumatology 2014, **5**: 23613 - http://dx.doi.org/10.3402/ejpt.v5.23613
